# Toward the Exploitation of Sustainable Green Factory: Biotechnology Use of *Nannochloropsis* spp.

**DOI:** 10.3390/biology13050292

**Published:** 2024-04-25

**Authors:** Davide Canini, Edoardo Ceschi, Federico Perozeni

**Affiliations:** Department of Biotechnology, University of Verona, 37134 Verona, Italy; davide.canini@univr.it (D.C.); edoardo.ceschi@univr.it (E.C.)

**Keywords:** *Nannochloropsis*, synthetic biology, genetic manipulation, genetic engineering

## Abstract

**Simple Summary:**

With the increase in the global population, food and energy production systems need to find immediately effective alternatives. There has been increased interest in microalgae thanks to their ability to grow exploiting photosynthesis with only water and simple nutrients, even derived from wastewater. Among microalgae, *Nannochloropsis* genus is one of the most interesting ones due to the capacity to accumulate “good” fatty acids, omega-3 and omega-6. Currently, the cultivation of microalgae is not often cost-effective, and strain domestication must be performed. Microalgae can be manipulated through metabolic engineering to increase their biomass productivity or to obtain high-value products that overcome the cultivation cost. Here, we review the main genetic manipulation tools and present some examples to explain the potential of *Nannochloropsis* spp. as a green cell bio-factory. The use of genetic engineering will finally bridge the gap between the probable and feasible use of microalgae.

**Abstract:**

Securing food, energy, and raw materials for a growing population is one of the most significant challenges of our century. Algae play a central role as an alternative to plants. Wastewater and flue gas can secure nutrients and CO_2_ for carbon fixation. Unfortunately, algae domestication is necessary to enhance biomass production and reduce cultivation costs. *Nannochloropsis* spp. have increased in popularity among microalgae due to their ability to accumulate high amounts of lipids, including PUFAs. Recently, the interest in the use of *Nannochloropsis* spp. as a green bio-factory for producing high-value products increased proportionally to the advances of synthetic biology and genetic tools in these species. In this review, we summarized the state of the art of current nuclear genetic manipulation techniques and a few examples of their application. The industrial use of *Nannochloropsis* spp. has not been feasible yet, but genetic tools can finally lead to exploiting this full-of-potential microalga.

## 1. Introduction

Microalgae are photosynthetic unicellular microorganisms able to assimilate CO_2_ through photosynthesis. Among all the organisms able to fix carbon dioxide, they have the significant advantage of being sustainable and not in competition with traditional agriculture. Their cultivation can occur in closed artificial systems, even using wastewater and industrial gasses, without the use of arable lands. Algal adaptation capacity and the possibility of growing them in fully controlled closed systems allows microalgae cultivation in geographic areas where traditional agriculture is ineffective [1,2] including planetary human habitation [3]. Microalgae are well known to be a low-cost and highly nutritive source that is useful for food or feed. Interesting nutritional profiles characterize most microalgae due to their high protein content as well as the accumulation of essential fatty acids (Ω-3 and Ω-6), vitamins, pigments, and antioxidants [4].

Microalgal biotechnology received significantly increased attention in recent years for the possibility of using these photosynthetic organisms as biorefinery for high-value products [5]. Microalgal metabolic engineering and their genetic manipulation are not a choice, and they are mandatory to make their exploitation feasible. Expensive cultivation costs do not allow the establishment of an effective industrial application for these microorganisms. In this scenario, microalgal genetic manipulation can pivot by generating mutants with suitable characteristics such as higher growth rate, biomass production, or metabolite accumulation. Moreover, using microalgae as a bio-factory for high-value products can offer a green sustainable way to produce valuable molecules and reduce cultivation costs.

**Figure 1:**
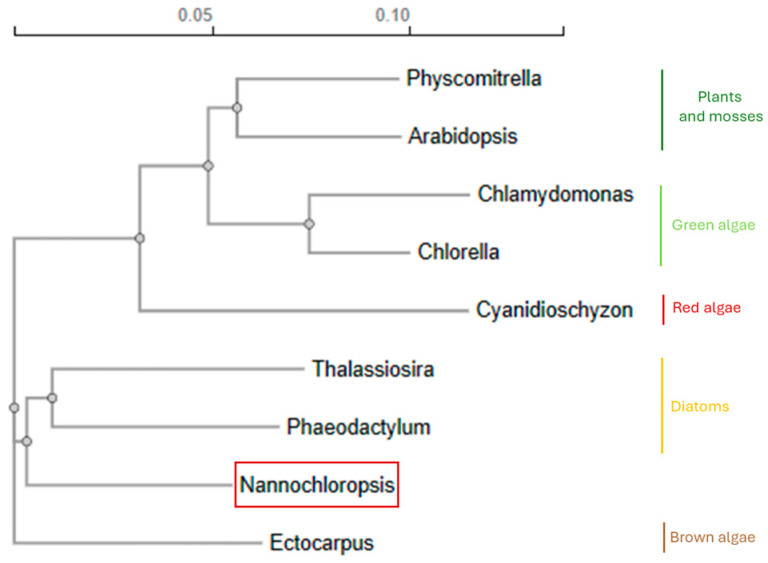
*Nannochloropsis* phylogenetic tree. Phylogenetic tree showing the evolution of *Nannochloropsis* spp. with respect to the other algae. The phylogenetic tree is obtained from the multiple sequence alignment of 18S ribosomal RNA gene (18S rDNA) using Clustal omega and neighbor-joining distance matrix. The following sequences from NCBI were used: *Physcomitrella patens* (AF126289.1), *Arabidopsis thaliana* (X16077.1), *Chlamydomonas reinhardtii* (AB511835.1), *Chlorella vulgaris* (X13688.1), *Cyanidioshyzon merolae* (XR_002461616.1), *Thalassiosira pseudonana* (MH545685.1), *Phaeodactylum tricornutum* (AJ269501.1), *Nannochloropsis* CCMP505 (U41050.1), and *Ectocarpus siliculosus* (L43062.1).

**Figure 2:**
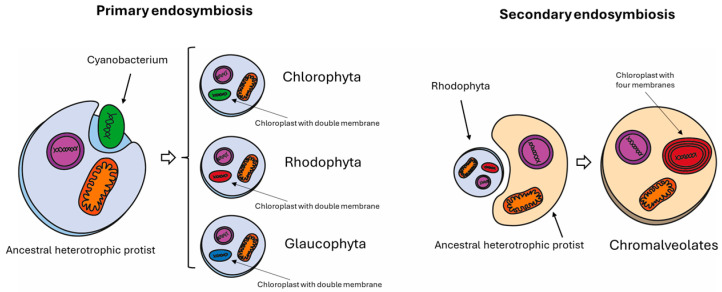
Diagram showing primary and secondary endosymbiosis events at the origin of *Nannocchloropsis* spp. The left panel shows the primary endosymbiosis at the origin of green algae (Chlorophyta), red algae (Rhodophyta), and Glaucophytes. In this process, a eukaryotic cell engulfs a prokaryotic cell that can undergo photosynthesis. As a footprint, the chloroplast of cells belonging to primary endosymbiosis is surrounded by a double membrane. By contrast, Chromalveolates, which include Cryptophyta, Haptophyta, Stramenopiles (or Heterokontophyta), and Alveolata, were originated by a secondary endosymbiosis. In this process, an eukaryotic cell engulfs another eukaryotic cell which has already engulfed a prokaryotic cell in its past (a Rhodophyte in that case). The Chromalveolate chloroplast, as a result of secondary endosymbiosis, is surrounded by four membranes.

*Nannochloropsis* spp. is unique among all microalgae, with peculiar attributes including a rapid growth rate in diverse environmental conditions and a high lipid content. The *Nannochloropsis* species belong to the *Eustigmatophyceae* (Figure 1), and their cells are spherical, non-flagellate, with a diameter of 2-8 µm [6]. Four membranes surround their chloroplast due to the secondary endosymbiosis event (Figure 2) [7]. Interestingly, the *Nannochloropsis* plastid is characterized by a continuum between the external chloroplast membrane and the nucleus envelope [8]. The single chloroplast occupies most of the cell volume. It contains a peculiar pigment content: only chlorophyll a (Chl a) is present, with a lack of other accessory chlorophylls, while violaxanthin, β-carotene, and vaucheriaxanthin are the most represented carotenoids under control conditions (100 μmol photons m^−2^s^−1^, 22 °C, 5% CO_2_ enriched air) [9].

The *Nannochloropsis* genus was historically composed of six known species: *Nannochloropsis gaditana* [10], *Nannochloropsis granulata* [11], *Nannochloropsis oceanica* [12], *Nannochloropsis limnetica* [13], *Nannochloropsis salina* [14], and *Nannochloropsis oculata* [14]. More recently, the results of phylogenetic analyses of the *Nannochloropsis* taxa on 18S rDNA revealed the presence of an additional *Nannochloropsis* species, *N. australis* [15]. In addition, the analysis on the plastid rbcL gene allowed researchers to revisit the phylogenesis of *Nannochloropsis* taxa, introducing a new sister genus called *Microchloropsis* and shifting *N. gaditana* and *N. salina* into that [15]. With this new classification, the *Nannochloropsis* genus comprises five species (*N. oculata*, *N. granulata*, *N. oceanica*, *N. limnetica*, and *N. australis*) while the *Microchloropsis* two (*M. gaditana* and *M. salina*). Moreover, *M. gaditana* is suggested to be classified as a strain of *M. salina*, sharing up to 98% of the chloroplastic genome and 97% of the mitochondrial one [16]. For coherency with the literature in this review, *M. gaditana* and *M. salina* will be referred to as *Nannochloropsis*. Except for *N. limnetica*, all *Nannochloropsis* species are found in marine environments [17].

*Nannochloropsis* can be cultivated on an industrial scale thanks to its robust growth and acquired popularity for its ability to accumulate high amounts of lipids and PUFAs such as arachidonic acid (AA, 20:4 n-6) and eicosapentaenoic acid (EPA, 20:5 n-3) [18,19,20,21]. *Nannochloropsis* can reach up to 69% of lipids on dry weight (DW) [22], 90% of TAG on total glycerolipids, and 42% of EPA on total fatty acids [23]. Its lipids include mainly the following glycerolipids: TAG, glycolipids monogalactosyldiacylglycerol (MGDG), digalactosyl-diacylglycerol (DGDG), and sulfoquinovosyl-diacylglycerol (SQDG); phospholipids: phosphatidylcholine (PC), phosphatidylethanolamine (PE), phosphatidylglycerol (PG), and phosphatidylinositol (PI); and non-phosphorus betaine lipid diacylglycerol-N, N, N-trimethylhomoserine (DGTS).

Oleaginous algae, including *Nannochloropsis*, produce only small quantities of TAG under optimal growth conditions [22]. Different stressing agents, such as chemical (nutrient starvation, salinity, and pH) or physical (light intensity and temperature) result in large amounts of TAG (up to 70% DW), accompanied by considerable alterations in lipid and fatty acid profiles [18,22,24,25,26,27]. The lipid accumulation increases in response to stressing growth conditions is a widespread trait among *Nannochloropsis* spp., but the amplitude of this phenomenon for each stimulus is strain-specific.

Light is essential to allow photosynthesis, but an excess of irradiation can irreversibly damage the photosynthetic apparatus, leading, in the worst case, to cell death [28]. *Nannochloropsis* grown in high-light saturating conditions shows a higher lipid, fatty acid, and carbohydrate content [18]. The increased lipid content is accompanied by a changed profile: eicosapentaenoic acid (C20:5) and arachidonic acid (C20:4) are reduced, while palmitic acid (C16:0) and palmitoleic acid (C16:1) are increased [18]. The changes occur as a consequence of the damage/resynthesis of the thylakoid membrane where EPA is mainly located. Moreover, besides intensity, light quality can modify the fatty acid accumulation and profile. The use of monochromatic red or blue light results in 1.2- and 1.4-fold higher percentages of saturated fatty acid accumulation than using white light in *N. gaditana* [29].

Temperature is another critical stimulus that can modify the fatty acid profile. Microalgae modulate membrane fluidity in response to temperature changes. In *N. salina*, it is reported that higher temperatures favor saturated fatty acid accumulation, while when the temperature decreases, the saturation level decreases as a consequence [30].

Lastly, nitrogen starvation is considered the stimulus with the highest response in triggering lipid accumulation [22]. In particular, TAG can be increased from around 0.5% to >30% of DW, and polar lipids can decrease from 5% to 1% of DW in *N. oceanica* [31,32,33]. EPA is mainly present in membrane lipids and, thus, decreases under nutrient depletion in response to membrane remodeling. The modulation of fatty acid composition and TAG, in response to nitrogen starvation, even if highly conserved, depends on *Nannochloropsis* species [31,33,34,35]. *Nannochloropsis* is mainly used as fish feed or a human food additive [10,36,37], and research on its exploitation for biofuel production is still ongoing. The aquaculture use of *Nannochloropsis* is also due to their ability to accumulate up to 20% DW of beta-glucans (βGs), which can stimulate the immune system of farmed fish [38].

*N. gaditana*, *N. salina*, *N. oceanica*, *N. granulata, and N. oculata* genomes were fully sequenced and can be used by the scientific community [39,40,41,42]. Nowadays, the genetic manipulation of *Nannochloropsis* is well established thanks to the optimization of several biotechnological tools, including CRISPR-Cas [43,44,45,46,47,48], or the possibility of performing homologous recombination in the safe harbor site [49] and to the availability of tens of genetic elements, ensuring an easy and fast generation of high-level expressing strains.

Here, we review the main biotechnological goals achieved in *Nannochloropsis*, focusing on technologies, limits, and potentials.

## 2. Biotechnological Tools

### 2.1. Genome Availability

As previously mentioned, the genomes of several *Nannochloropsis* species were fully sequenced and annotated [39,40,41,42,50,51]. These nuclear genomes have an approximative size of ~29 Mb, a reduced intron content, short intergenic regions, and over 10.000 genes [39,51]. Moreover, the chloroplast and the mitochondria genomes are ~117.5 Kb and ~38 Kb, respectively [42]. Genome assembly is well supported by transcriptome data under different conditions but mainly focuses on nutrient starvation, considering the increased lipid accumulation in this condition [35,52,53,54,55,56].

### 2.2. Transformation Methods

Since 2008, when the first successful attempt to transform the nuclear genome of *Nannochloropsis oculala* was reported [57], the scientific community has efficiently established genetic manipulation tools, including several transformation methods. However, the first report on an efficient transformation by means of electroporation on *Nannochloropsis* whole cells was published in 2011 [58]. Electroporation-mediated methods are well established and broadly used nowadays, allowing the breaking of the rigid cell wall and efficiently introducing exogenous genetic material [59]. Other nuclear transformation methods, such as agrobacterium-mediated transformation [60] or particle bombardment [61,62], have been proposed, but their use remains limited and poorly reported.

### 2.3. Promoters

Efficient transgene expression relies on regulatory elements such as promoters, enhancers, and terminators. The number of promoters available for *Nannochloropsis* is far lower with respect to other model microalgae or plants. However, several promoting elements, including constitutive, inducible, and bidirectional, have been identified and can be used by genetic engineering community (Table 1). For transgene expression, strong endogenous promoters are usually used. Some examples of broadly used DNA regions to initiate transcription are the sequence from the gene encoding β-tubulin [63,64], lipid droplet surface protein (LDSP) [39], ubiquitin extension protein (UEP) [64], violaxanthin/chlorophyll a-binding protein 1 (VCP1) [58], elongation factor (EF) [56,65], and the Heat Shock Protein (HSP) [63]. These sequences can guarantee a high-level transcription and are widely used for *Nannochloropsis* metabolic engineering. Two other promoters have acquired popularity for one peculiar characteristic: these are the VCP2 promoter [58,66] and the intergenic region between two ribosomal subunit genes (Ribi) [58,67], which are bidirectional [58,67]. Bidirectional promoters offer several advantages: integrating two genes that share the same promoter reduces the length of the expression cassette and halves the number of transformation events required. This increases the transformation efficiency and decreases the time needed to obtain the desired strains. The expression level is, however, not equal, with slight differences in the two opposite directions [68].

The last class of promoters available for *Nannochloropsis* are the inducible ones. The sequences from sulfoquinovosyl diacylglycerol synthase 2 gene (SQD2) [69] or nitrate reductase gene (NR) [70] can be used to modulate gene expression in response to phosphorus or nitrate concentration. Inducible promoters offer the possibility to accumulate products even if they are cytotoxic for the cells. The accumulation of the enzymes responsible for toxin catalysis could not happen in the absence of the inductor. The mechanisms by which SQD2 and NR promoters induce gene expression are different: the first starts the transcription in the absence of phosphorus, while the second starts in the presence of nitrate. Both promoters, as any inducible promoter, show a basal expression level that, if very low, needs to be considered [71]. An interesting application of inducible promoters was carried out by de Grahl and colleagues in *Nannochloropsis oceanica* [71]. They proposed the auto-induction of gene expression by switching the nitrogen source into the growth medium from ammonium to nitrate. *N. oceanica* utilizes preferentially ammonium rather than nitrate because the first can be directly inserted in the pathways for amino acid synthesis. Moreover, they demonstrated that the NR gene expression is suppressed in the presence of ammonium. Considering that, they developed an auto-inducible medium using a precise amount of ammonium in the presence of nitrate; once ammonium is wholly utilized, the expression of genes driven by NR can start. By calibrating the amount of ammonium, transgene expression can be modulated in time and optimized according to algal growth.

Recently, several constitutive as well as inducible promoters were tested by evaluating their ability to drive YFP expression. The results demonstrated that the promoting sequences from the EF, α-tubulin, and NR genes were able to ensure the highest reporter expression with respect to the promoters from VCP1, LDSP, and violaxanthin/chlorophyll a-binding like protein (VCP-L) [71]. It is essential to underscore that while the promoters from EF and TUB are constitutive, the one from NR is inducible and requires nitrate-containing growth media. In addition, the synergy between promoters and the initial part of their open reading frame sequences was underlined. Promoters from EF and NR showed the highest YFP expression if the sequence was extended by 42 bp [71].

**Table 1:** List of available promoters for *Nannochloropsis* spp.

Promoter	*Nannochloropsis* Strain	References
Lipid droplet surface protein (LDSP)	*N. oceanica* CCMP1779	[39,56,72]
	*N. oceanica* IMET1	[49]
β-tubulin	*N. gaditana* CCMP526	[73]
	*N. oceanica* IMET1	[46,63]
	*N. salina* CCMP1776	[64,74,75]
Ubiquitin extension protein (UEP)	*N. gaditana* CCMP526	[73]
	*N. salina* CCMP1776	[64,74]
Violaxanthin/chlorophyll a-binding protein 1 (VCP1)	*N. oceanica* IMET1	[44,46,49,58,76]
Violaxanthin/chlorophyll a-binding protein 2 (VCP2)	*N. oceanica W2J3B*	[58]
	*N. oceanica CCMP1779*	[66]
	*N. oceanica IMET1*	[63]
Elongation factor (EF)	*N. oceanica* CCMP1779	[56,65]
Heat Shock Protein (HSP)	*N. gaditana* CCMP526	[73,77]
	*N. oceanica* IMET1	[63]
Ribosomal subunits (Ribi)	*N. oceanica* CCMP1779	[56]
Extrinsic protein in photosystem II (EPPSII)	*N. gaditana* CCMP526	[77]
ATPase	*N. gaditana* CCMP526	[77]
Sulfoquinovosyl diacylglycerol synthase 2 (SQD2)	*N. oceanica* NIES-2155	[69]
Nitrate reductase (NIT)	*N. gaditana* CCMP526	[70]

### 2.4. Selection Markers and Reporters for Gene Expression

The co-integration of a selectable marker and the gene of interest is a typically used strategy in genetic manipulation, not only for microalgae. Conferring the ability to grow in the presence of selective agents, this strategy allows the isolation of cells that have integrated the exogenous DNA. The bleomycin resistance protein gene (*Sh ble*) conferring resistance to zeomycin is the most utilized selection marker in *Nannochloropsis* spp. Hygromycin-B phosphotransferase conferring resistance to Hygromycin, aminoglycoside 3′-phosphotransferase which allows growth on G418 medium, and nourseothricin acetyltransferase conferring resistance to Nourseothricin are other selection markers used [68]. The available selection markers for *Nannochloropsis* spp. are reported in Table 2.

It is essential to underscore that not all antibiotics have the same effect on all *Nannochloropsis* species, and the range of concentration can be highly different. Vieler and coworkers [39] compared the impact of 10 antibiotics in four *Nannochloropsis* species. Six (Rifampicin, Benomyl, Nystatin, Spectinomycin, Ampicillin, and Chloramphenicol) were reported not to cause negative growth effects at any tested condition. For that reason, their use as selection markers is not possible. On the contrary, zeomycin could completely abolish algal growth using the same concentration (5 μg/mL) in all tested species. The other antibiotics showed no evenly distributed dose effect among the different *Nannochloropsis* species. For example, Hygromycin B worked in *N. oceanica*; doubling the antibiotic dose allowed for selection also in *N. granulata* and *N. gaditana*, too, but even higher concentrations had no effects in *N. salina*.

A different use of antibiotics was recently proposed by Zhang and colleagues [78]. The antibiotic zeomycin was used in *Nannochloropsis oceanica* as a 48 h transformation pre-treatment at 0.2 μg/mL dose. Antibiotic exposure causes random double strain breaks in microalgal cells, leading to a 10- to 40-fold higher transgene integration [78].

Even though homologous recombination (HR) in *Nannochloropsis* has been reported [58], the efficiency of this type of integration is strain-specific and highly influenced by growth conditions. Most transgenes are randomly integrated during the nuclear transformation into the host genome, and the homologous recombination efficiency is poor. In addition, HR can be a valuable tool to generate knockout mutants or in the presence of specific sites in the genome to ensure a high expression level (see Section 2.4), but it is not strictly required to perform genetic engineering and requires the identification of a “neutral” site in order not to interfere with algal metabolism. The random insertion leads to differences in integration sites that greatly influence the expression via the “position effect”, with high variability among transformants. Therefore, target proteins are typically fused to fluorescent reporters to allow the identification of lines with the highest transgene expression. Creating chimeras with proteins of interest and fluorescent reporters provides a high throughput generation of expressing lines. Moreover, besides the fast identification of the highest expressing lines, fluorescent proteins allow the performance of immunodetection or purification in the absence of specific antibodies. Nevertheless, it is essential to underline that the presence of fluorescent proteins fused to the one of interest can interfere with protein folding and, in some cases, affect protein activity. To reduce this risk, linkers (such as the GSG linker) are usually placed between the two proteins. In contrast, fluorescent proteins can be easily used in basic research because they are simply foldable, stable, and fast-detectable. In that case, reporters are largely used to compare different promoters, to optimize or set transformation methods, or to evaluate target peptide activity.

Starting from the *Aequorea victoria* green fluorescent protein (GFP) [79], several variants, such as Yellow FP and Cyan FP, with a different absorption/emission, were developed [56]. In addition to those, researchers can use SfCherry or the DsRed variant tdTomato derived from *Discosoma* spp [45,61]. The most commonly used fluorescent reporters with their absorption and emission spectra are shown in Table 3.

Moreover, luminescent proteins or chromoproteins are also used in *Nannochloropsis*. For example, the ultra-bright NanoLuciferase was used in *N. oceanica* [80], while the purple chromoprotein (shPCP) was used in *N. oculata* for transformant screening [81].

### 2.5. Genetic Knockout Strategies

**Figure 3:**
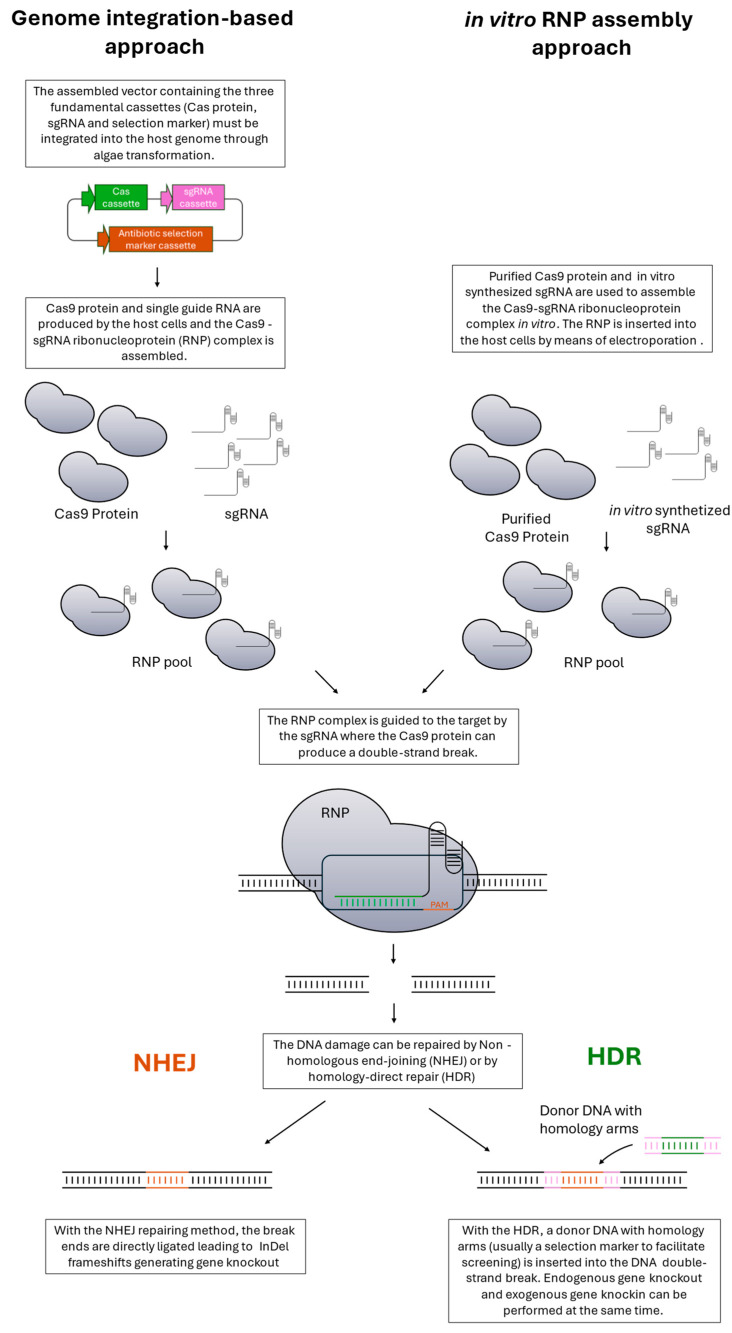
CRISPR/Cas9 mechanism. The main components are an RNA-guided Cas9 endonuclease and a single guide RNA (sgRNA). The genome integration-based approach (left side) requires the construction of a vector carrying three different cassettes: one encoding for the Cas9 protein, the second for the sgRNA, and the third for antibiotic resistance. Once the vector is stably integrated into the host genome, Cas9 protein and sgRNA are synthesized through cell machinery. On the contrary, the in vitro ribonucleoprotein (RNP) assembly approach (right side) requires Cas9 purified protein, usually from *E. coli*, and in vitro synthesized sgRNA. Both in the integration and in the in vitro RNP assembly, Cas9 protein and sgRNA are assembled to form Cas9 (RNP) with the ability to bind and cleave target DNA [92]. The main difference is that in the former, the RNP complex is assembled by the cell itself, while in the latter, RNP is in vitro assembled and later inserted into the cell. Single guide RNA drives the RNP to the targeted site that must be close to the protospacer adjacent motif (PAM) [93]. The cleavage carried out by Cas9 is a double-strand blunt break (DSB). Once the DSB is introduced in the target gene by Cas9 nuclease activity, the cell has two alternatives for the repair: non-homologous end-joining (NHEJ) or homology-directed repair (HDR) [93]. The first can introduce insertion or deletion at cleavage sites, generating a frameshift into the gene open reading frame. The latter can introduce exogenous DNA at the target site by using a homologous DNA repair template. In the HDR, usually, as donor DNA, the resistant marker cassette is used to allow transformant screening.

Gene downregulation or disruption is a common strategy to understand metabolic aspects or to generate backgrounds with tailored characteristics for engineering. The haploid nature of the *Nannochloropsis* genome facilitates the obtainment of mutants with the desired phenotype with respect to diploid or polyploid organisms. Commonly, proteins are translated starting from only one gene, causing perturbation in cell metabolism according to the importance of the enzyme when it is disrupted. Forward genetic methods (i.e., random mutagenesis or insertion) can thus be used in *Nannochloropsis* to obtain mutants with the desired phenotype and offer the possibility to identify key genes useful for reverse genetics. However, using random insertion or mutagenesis to generate knockout mutants has a low success rate. The insertion of the donor DNA, which must happen into an exon, is a rare event. On the other hand, the use of mutagenesis to knock out a gene presents even more constraints because the mutation can be silent or can lead to an amino acid substitution not being able to perturb the protein folding/activity. In both cases, identifying positive individuals is time-consuming because it requires the screening of many cells and only sometimes does it lead to a positive result. For that reason, targeted methods are preferred, but all reverse genetic approaches require a knowledge of genomes and pathways. Historically, the first targeted method to manipulate specific gene expression was RNA interference (RNAi). This powerful tool allows the silencing of specific genes, but their expression is not completely abolished. RNAi was largely used in *Nannochloropsis* to produce stains with reduced gene expression [75,82,83]. With the sequencing of genomes and the emergence of CRISPR/Cas systems, the generation of knockout mutants in *Nannochloropsis* became feasible and primarily applied. RNA-guided engineered nucleases (RGENs) have been largely applied to microalgae and rapidly superseded other targeted editing methods, such as zinc finger nucleases and transcription-activator-like effector nucleases. The optimization of RGEN methods, initially based on the *Streptococcus pyogenes* type II CRISPR/Cas9 system (SpCas9), made the generation of knockout mutants in *Nannochloropsis* feasible and easily accessible. The system uses a Cas9 nuclease and a single guide RNA to induce DNA breaks into the genome; during the repair of these breaks, random insertion/deletion (InDel) or exogenous DNA insertion by HR can occur, generating knockout mutants. For a more detailed description of the mechanism, see Figure 3. The first reported attempt dates to 2016, when Wang and colleagues successfully knocked out the nitrate reductase gene in *N. oceanica* using the CRISPR/Cas9 system [46]. Since then, several improvements in the techniques have been made to increase the knockout efficiency [43,44,45,46,47]. The ease and low cost of this technology resulted in a significant diffusion of RGENs CRISPR/Cas systems. However, the constitutively stable expression of Cas protein into the host genome presents few constraints. One of the already reported problems in this technique is the cytotoxicity of Cas once integrated into the algal genome. Previously identified and reported in the model microalga *Chlamydomonas reinhardtii*, this cytotoxic effect results in an extremely low targeted mutagenesis efficiency [84]. To overcome that, DNA-free RGEN-based methods are proposed for several organisms [85,86,87,88,89]. These approaches are based on in vitro preassembled Cas-gRNA ribonucleoproteins. As mentioned before, the DNA-free approach reduces the off-target and cytotoxic effects because the Cas protein is only transiently present. Furthermore, the technology does not require codon optimization or specific promoters being rapidly transferrable between different organisms. The preassembled Cas-sgRNA ribonucleoprotein-based method was also successfully used in *Nannochloropsis oceanica* by inserting the complex using electroporation [44]. Two other approaches to produce gene knockout without a stable Cas integration into genome must be cited. First, Poliner and coworkers developed an episomal-based system exploiting the CEN/ARS6 region from *S. cerevisiae* [48]. The main advantage of this technique is to represent a marker-free system; once the selective pressure is removed, the episomal is lost and no longer present in microalgae. With a similar episomal-based system exploiting the Cas12 activity, Naduthodi and coworkers recently performed multiple gene editing in *N. oceanica* [45]. In the same paper, the possibility of performing CRISPR-mediated interference was also presented [45]. Although Cas9 was historically the first Cas used and is currently the most used one, recently, Cas12a (formerly called Cpf1), a member of class V, has gained global attention as an interesting alternative. Cas12 presents a more minimalistic system than Cas9 since the latter consists of two nuclease domains that produce a blunt end cleavage, whereas Cas12a has a single domain that generates staggered ends on both the target and the non-target DNA strands (Naduthodi et al., 2018; Swarts et al., 2017). In addition, the protospacer adjacent motif (PAM) sequence recognized by Cas9 is placed downstream of the spacer sequence on the non-target strand; the Cas12a PAM sequence is located upstream of the spacer. For these reasons, Cas12a exhibits higher efficiencies than Cas9 in generating mutants through HDR and fewer off-target effects [45]. Lastly, it is important to cite that homologous recombination (HR), even with the abovementioned limits, is another possibility for generating knockout mutants [90,91].

### 2.6. Multiple Gene Expression

The possibility of overexpressing multiple genes represents one of the main desirable goals for the metabolic engineering scientific community. Metabolic engineering is the use of genetic manipulation to modify the metabolic network of cells to produce desirable products or to tune phenotype traits. Usually, manipulating a single gene does not lead to the accumulation of the desired product, and a complex rearrangement of the overall metabolic pathway needs to be performed by acting on several enzymes. The typical approach involves the use of sequential individual transformations, exponentially increasing transformation markers and fluorophores, and taking the time to generate expressing lines. Moreover, besides the limited number of markers available, the promoter pool is limited too: using the same promoting regions several times can lead to regulatory mechanisms decreasing the transcription level. Unless specific manipulations in that direction are made, the number of transcription factors able to bind a specific region is limited. Using the same promoter region to express several genes could lead to “compensative” mechanisms in which the last added gene is transcribed at the expense of the others already present. For these reasons, the insertion of multiple genes is a challenging topic. The first possibility, also exploited in other organisms, is to use the self-cleaving 2A peptide [94]. This peptide is known for generating independent proteins starting from the same transcript. This reduces the number of promoters/terminators and transformation events without generating fusion proteins. Although the 2A peptide technology has already been used in *N. oceanica* [80] and *N. salina* [95], the cleavage efficiency is less than 50%. As an alternative, Poliner and coworkers recently developed a high-capacity gene stacking toolkit to ensure the expression of several genes using a single vector, and thus one transformation event and one selection marker [68]. Using this new technology, which takes advantage of Gateway cloning and the bidirectional promoter Ribi, researchers could co-express three reporter genes (GFP, Flux, and Nlux) in addition to antibiotic resistance.

### 2.7. Genomic Safe Harbor as a Method of Maximizing Transgene Expression

**Figure 4:**
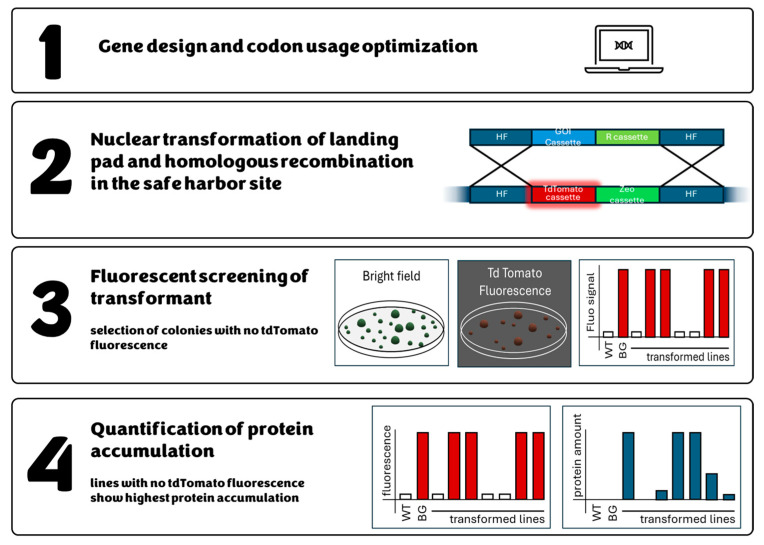
Screening pipeline for transformants using the “safe harbor” system. Briefly, genes of interest are in silico designed, optimizing codon usage and adding homologous recombination sequences (**1**). *Nannochloropsis* landing pad strain nuclear transformation is performed by means of electroporation, and homologous recombination (HR) occurs in the safe harbor site (**2**). td Tomato fluorescence can be used to identify lines in which homologous recombination occurred. WT shows no fluorescence, background landing pad strains (BG) show maximum fluorescence, while the transformed lines have or do not have fluorescence according to the occurrence of HR (**3**). Protein quantification allows quantifying the protein of interest; lines showing no tomato fluorescence, in which HR occurs in the safe harbor site, show the highest protein accumulation; other transformed lines show no or lower protein accumulation due to random insertion into the genome (**4**).

Using microalgae as a bio-factory for producing metabolites has yet to be feasible and sustainable. This is mainly due to the poor transgene expression in microalgal hosts, primarily caused by the aforementioned “position effect”. Recently, Südfeld and coworkers identified the rDNA cistron on chromosome 3 of *N. oceanica* as a genomic safe harbor for transgene expression by Pol I [49]. A new expression system based on a chimeric internal ribosome entry site (IRES) and a landing pad strain allows a high-throughput generation of expressing lines. The system, summarized in Figure 4, uses a landing pad strain in which the fluorescent gene tdTomato is inserted into the safe harbor site. A gene of interest can be inserted in this site by homologous recombination, removing the fluorescent protein gene. Thus, this system has two main advantages: it ensures the highest transgene expression already reported and allows the fast and easy screening of transformants. 

### 2.8. Chloroplast Genome Manipulation

The majority of genetically engineered organisms are generated by integrating transgenes in their nuclear genome. However, photosynthetic organisms also offer the opportunity for chloroplast transformation. Integrating transgenes into the chloroplast genome has several advantages, including the possibility of performing homologous recombination, avoiding position effects, and the absence of silencing processes in this organelle [96]. Chloroplast transformation has been reported in higher plants (see [97] for a review), but the plastid microalgal biotechnology is well established only in *C. reinhardtii* [98,99,100]. Only two reports showed chloroplast engineering in *Nannochloropsis* [101,102]. Gan and coworkers successfully integrated into the chloroplast genome of *N. oceanica* the GFP-encoding gene, leading to a clear GFP protein accumulation and fluorescence [101]. In addition, Cui et al. engineered the plastid genome of *N. gaditana* by inserting two codon-optimized antimicrobial peptide-coding genes in a polycistronic sequence. Besides the fact that, to our knowledge, these are the only successful attempts, the reported transformation efficiency is low and must be improved to feasibly exploit this technology. However, at the same time, they show that also in *Nannochloropsis*, the chloroplast is a suitable compartment for heterologous protein expression.

## 3. Biotechnological Exploitation: A Few Examples

Metabolic engineering in *Nannochloropsis*, albeit feasible, is rarely used with respect to other microalgae such as *Chlamydomonas* [103]. The potential of *Nannochloropsis* as a host for metabolite production is high and needs to be explored with the abovementioned genetic tools. Here are several examples of genetic manipulation approaches aimed at generating strains with suitable characteristics.

### 3.1. Lipids

Our current knowledge of the lipid pathways in *Nannochloropsis* spp. is limited, and the same applies to the evolutionary close groups of diatoms and brown algae. Nevertheless, these pathways are strongly conserved among microalgae and plants. Therefore, it is possible to take advantage of our understanding of the model organisms *Chlamydomonas reinhardtii* and *Arabidopsis thaliana*.

**Figure 5:**
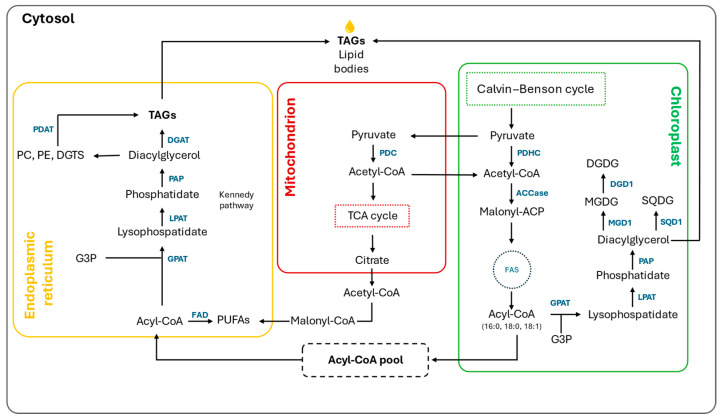
*Nannochloropsis* lipid metabolism. The main characterized enzymes are written in dark blue. Abbreviations: ACCase, acetyl-CoA carboxylase; DGAT, diacylglycerol acyltransferase; DGD1, digalactosyldiacylglycerol synthase; FAD, fatty acid desaturase; FAS, fatty acid synthase; FAE, fatty acid elongase; GPAT, glycerol 3-phosphate acyltransferase; LPAT, lysophosphatidic acid acyltransferase; MGD1, monogalactosyldiacylglycerol synthase; PAP, phosphatidic acid phosphatase; PDAT, phospholipid:diacylglycerol acyltransferase; PDHC, pyruvate dehydrogenase complex; SQD1, UDP-sulfoquinovose synthase 1; CoA, coenzyme A; DGDG, digalactosyldiacylglycerol; G3P, glycerol 3-phosphate; MGDG, monogalactosyldiacylglycerol; SQDG, sulfoquinovosyldiacylglycerol; TAG, triacylglycerol; PUFA, polyunsaturated fatty acid.

The de novo synthesis of fatty acids occurs mainly in the chloroplast, and, subsequently, they are exported to the ER. In contrast, the glycerolipids are assembled both in the chloroplast and ER (where the pathway is known as the Kennedy pathway). The chloroplast and ER contain similar enzymes: acyltransferases (GPAT, LPAAT), phosphatases (PAP), and synthases (CDS, PGPS). On the contrary, different acyl donors, acyl-acyl-carrier protein in the chloroplast and acyl-coA in ER, mediate the acylation step. Fatty acid modification, such as desaturation, primarily occurs in chloroplast and ER membranes, while elongation happens in the chloroplast and ER through acyl-ACP and acyl-coA, respectively. Lipid remodeling via acyltransferases and lipases is fundamental for fatty acid exchanges between membrane lipids and TAG [104]. An overview of *Nannochloropsis* lipid metabolism is reported in Figure 5.

To increase the oil production in *Nannochloropsis* spp. several metabolic engineering strategies have been used, including transcription factors, enzymes involved in lipid pathways, and enzymes involved in other pathways. The overexpression of the bZIP1 transcription factor in *N. oceanica* increased intracellular lipid content by 100% and enabled unprecedented lipid secretion [105]. The knockout of APETALA2-like transcription factor in *N. oceanica* increased neutral lipid content by 40% through the upregulation of genes involved in fatty acid biosynthesis, the CBB cycle, and sugar metabolism [76]. Moreover, the overexpression of acyl-CoA: diacylglycerol acyltransferase (DGAT1) in *N. oceanica* increased TAG and TFA by 50% and 14% of DW, respectively [82], while the upregulation of DGAT2 showed a slight effect on TAG, and DGAT5 increased TAG fatty acids on TFA from 10 to 40% [65]. Lastly, the overexpression of a putative endogenous gene for malonyl CoA: ACP malonyltransferase (MCMT) in *N. oceanica* improved lipid content by 30% of DW [106].

Lipid content was also tuned by acting on enzymes not directly involved in their biosynthesis. The overexpression of a deoxy-xylulose 5-phosphate synthase (AtDXSoe3) from *A. thaliana* in *N. oceanica* increased, under high light, total lipids and TAG on DW by 60% and 80%, respectively [107]. Moreover, mutation on a DGDG synthase in *N. gaditana* increased FAME content from 100 to 160 mg/g DW [26]. Lastly, the overexpression of malic enzyme (ME) in *N. salina* increased biomass and FAMEs on DW by 15% and 30%, respectively [108].

Recently, few efforts have been made to increase EPA content. The overexpression of Δ5 desaturase in *N. oceanica* increased EPA content from 17 to 22% of total lipids, and the co-expression of Δ5 and 12 desaturases enabled it to reach 25% of EPA [80]. Moreover, the co-expression of a DGAT from *C. reinhardtii* (CrDGTT1) and an endogenous Δ0 elongase (Δ0-ELO1) increased EPA content in TAG up to 7.3%, which was 5.9-fold higher than WT [109].

### 3.2. Carotenoids and Other Pigments

As mentioned, the *Nannochloropsis* chloroplast evolved from secondary endosymbiosis and contains only Chlorophyll a in its light-harvesting complexes. Violaxanthin, vaucheriaxanthin, and β-carotene are the most abundant carotenoids, with neoxanthin, antheraxanthin, zeaxanthin, canthaxanthin, and astaxanthin accumulated only in traces [110]. However, changing nutritional and environmental factors can vary the carotenoid and chlorophyll content.

Carotenoids and chlorophylls in *Nannochloropsis* are extensively studied. The fundamental role in the light harvesting, energy dissipation, and photosynthetic protein folding of carotenoids is broadly reported in all photosynthetic organisms. Their investigation is, thus, essential to disentangle molecular mechanisms and to increase the general knowledge about *Nannochloropsis*. On the other hand, biotechnological approaches aimed at manipulating pigment content can be performed to generate strains with tailored pigment content.

For example, the overexpression of the LHCYB (lycopene beta cyclase) gene in *N. oceanica* increased carotenoid content, especially β-carotene [110]. In another case, the knockout of the VDE gene generated by insertional mutagenesis allowed the reduction in the zeaxanthin content and, at the same time, demonstrated the fundamental role of this pigment for light energy dissipation and ROS protection [111]. Moreover, Dauterman and coworkers generated a VDL (violaxanthin-de-epoxidase-like) mutant in *N. oceanica* with an increased violaxanthin accumulation [112]. In addition, Liu and colleagues obtained the same result with a different strategy: they overexpressed in *N. oceanica* the ZEP enzymes responsible for the epoxidation of zeaxanthin to violaxanthin. This manipulation led the microalgae to increase violaxanthin accumulation at the expense of zeaxanthin [113]. Moreover, they also demonstrated that, on the contrary, the suppression of the ZEP gene results in a decrease in violaxanthin and an increase in zeaxanthin contents [113]. Gamma-rays were also used to increase the violaxanthin content; random mutagenesis in *N. oceanica* allowed for the identification of a mutant with an increased violaxanthin content and an enhanced biomass production [114]. Efforts were also made to improve the astaxanthin content of *Nannochloropsis*. By means of chemical mutagenesis, Cecchin and coworkers isolated a new *Nannochloropsis gaditana* strain characterized by increased lipid and ketocarotenoid accumulation [115].

Chlorophylls are also an interesting target for metabolic engineering. Koh and colleagues acted on chlorophyll biosynthesis to increase the productivity of microalgae biomass. *Nannochloropsis* spp. completely lack accessory chlorophylls, but they demonstrated that the heterologous expression of chlorophyllide a oxygenase from *Chlamydomonas* (*CrCAO*) in *N. salina*, catalyzing the two-step oxygenation of chlorophyllide a to chlorophyll b, leads to positive effects, increasing the growth rate [116].

### 3.3. Other High-Value Products and Biotechnological Manipulation

*Nannochloropsis* spp. are widely used in aquaculture. The biotechnological production of high-value products also reflects this application. Chen and coworkers were able to engineer *N. oculata* to produce fish growth hormone, which promotes the growth of Tilipia larvae [57]. *N. oculata* was also used for antimicrobial peptide synthesis; the bovine lactoferricin produced in this host increased the survival rate of fish after *Vibrio parahaemolyticus* infection [117]. Lastly, as a model vaccine for fish immunization, the viral surface protein 2 (VP2) of a pathogenic fish virus that causes infectious pancreatic necrosis was produced in *N. oceanica* [118].

Another important metabolite was produced by exploiting the safe harbor pipeline. A camelid-type VHH antibody was successfully synthesized in *N. oceanica* [49].

Another class of molecules that can be produced in microalgae and with a broad range of biotechnological applications, such as flavoring, biopharmaceuticals, and cosmetics, are terpenoids {Masyita, 2022 #710}. *C. reinhardtii* was broadly used as chassis for their production [119,120,121,122]. In *N. oceanica*, Du and colleagues produced the diterpene casbene, widely used as an intermediate in the pharmaceutical industry [123]. Despite terpenoids’ potential, no other cases are reported for them in *Nannochloropsis*.

The reduction in cultivation costs is strictly connected to increasing microalgal growth. Increasing biomass production is one of the main desirable goals to make microalgae use cost-effective and feasible. Perin and colleagues screened EMS-obtained *N. gaditana* random mutants for better photosynthetic performance. They identified mutants in which photosynthetic antennae, or the heat dissipation mechanism, are affected with higher growth in a photobioreactor [124]. In addition, Arora and coworkers identified, using the same technique, two mutants exhibiting higher carbohydrate and pigment productivity [125]. Lastly, with a completely different approach, the basic helix-loop-helix transcription factor (NsbHLH2) overexpression improved biomass and lipid production [126].

## 4. Conclusions

The state-of-the-art engineering strategies are summarized in this review, and the examples reported demonstrate the potential of *Nannochloropsis* as an emerging green bio-factory for valuable bioproducts. However, further research is necessary to enrich the number of available genetic blocks, increase production titers, and allow industrially relevant applications. The use of *Nannochloropsis* as an algal chassis still has a long road ahead but the potential for exploitation is high and the possibility is concrete with the latest advantages in genetic manipulation tools.

## Figures and Tables

**Table 2:** List of available selection markers.

Protein	Conferring Resistance to	Reference
Bleomycin resistance protein (*Sh ble*)	Zeomycin	[58]
Hygromycin-B phosphotransferase (AphVII)	Hygromycin	[39]
Blasticidin-S deaminase (BSD)	Blasticidin	[68]
Nourseothricin acetyltransferase (NAT)	Nourseothricin	[68]
Aminoglycoside 3′ phosphotransferase	G418	[68]

**Table 3:** Most commonly used fluorescent reporters in *Nannochloropsis* spp. for screening. Excitation and emission maximum wavelength and brightness are reported. Data from FPbase (www.FPbase.org) (accessed on 24 March 2024).

Name	Ex λ	Em λ	Brightness	Excitation/Emission Spectra
Clover (GFP)	505	515	84.36	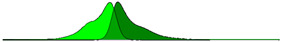
mVenus (YFP)	515	527	66.56	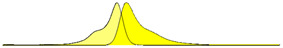
mCerulean (CFP)	433	475	34.8	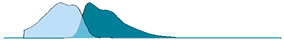
sfCherry	589	610	17.47	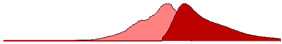
tdTomato	554	581	95.22	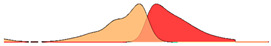

## Data Availability

No new data were created or analyzed in this study. Data sharing is not applicable to this article.
